# Reliability and quality of hypothyroidism videos on TikTok and Bilibili: A cross-sectional content analysis in China

**DOI:** 10.1097/MD.0000000000047523

**Published:** 2026-02-06

**Authors:** Yaqin Hu, Zongyou Cheng, Hongmin Zhu

**Affiliations:** a Department of Endocrinology, Jiangxi Provincial People’s Hospital, The First Affiliated Hospital of Nanchang Medical College, Nanchang, China; b Department of Cardiology, Jiangxi Provincial People’s Hospital, The First Affiliated Hospital of Nanchang Medical College, Nanchang, China.

**Keywords:** Bilibili, Global Quality Score, hypothyroidism, modified DISCERN, social media, TikTok

## Abstract

Hypothyroidism is a common endocrine disorder that significantly impacts patients’ quality of life. In recent years, short-video platforms have become an important source of health information for the public. This study aimed to evaluate the content, quality, and reliability of hypothyroidism-related videos on TikTok and Bilibili. We searched for videos related to “hypothyroidism” on TikTok and Bilibili and included the top 150 videos based on comprehensive ranking, excluding irrelevant, duplicate, advertisement, and course-related videos. Extracted variables included video duration, number of likes, collections, comments, shares, uploader type, and content themes. The Global Quality Score and modified DISCERN (mDISCERN) tools were used to assess each video. Mann–Whitney *U* tests and Kruskal–Wallis tests were used to compare differences, and Spearman correlation analysis was performed to examine associations between engagement metrics and video quality. A total of 270 videos were included. Video content primarily focused on treatment (62.2%) and symptoms (60.0%), whereas prevention (7.0%) and epidemiology (4.1%) were notably underrepresented. Videos on Bilibili were longer but had lower engagement (*P* < .05), while TikTok videos had higher mDISCERN scores. Videos uploaded by specialists received the highest Global Quality Score and mDISCERN scores (*P* < .05). Engagement metrics were strongly intercorrelated (*P* < .05), but showed no significant association with video quality (*P* > .05). Video length demonstrated a weak correlation with quality (*P* < .05). This study revealed that hypothyroidism-related short videos generally have incomplete content structures, particularly with insufficient coverage of prevention and epidemiology. The overall quality and reliability were suboptimal, with videos by specialists demonstrating higher quality. Future efforts should encourage greater participation of specialists in short-video health education content creation, and platforms should strengthen content oversight and optimize algorithms to enhance the visibility of high-quality video content.

## 1. Introduction

Hypothyroidism is a common endocrine disorder that poses a significant burden to public health. It is not only prevalent but also closely linked to cardiovascular disease, metabolic dysregulation, renal impairment, cognitive and mood disturbances, adverse reproductive and pregnancy outcomes, and increased all-cause mortality.^[1–3]^ Hashimoto thyroiditis is the most common cause of hypothyroidism. Its pathogenesis is primarily driven by chronic inflammation and autoimmunity, involving diverse immune cell populations, cytokines, and inflammatory signaling pathways.^[4,5]^ Studies have shown that multiple serum inflammatory markers are significantly elevated in patients with Hashimoto thyroiditis compared with healthy controls.^[6,7]^ Because the clinical manifestations of hypothyroidism are nonspecific, public awareness of symptoms, risks, and treatment remains limited, contributing to a high underdiagnosis rate, treatment delays, worsened complications, and greater socioeconomic burden.^[8,9]^ High-quality health communication is essential for enhancing disease literacy and recognition of healthcare-seeking pathways, thereby facilitating the early detection, standardized management, and improved health outcomes of hypothyroidism.^[8,10]^

In recent years, the rapid diversification of social media platforms such as TikTok and Bilibili has markedly accelerated the dissemination of public health information in China and globally.^[11]^ With hundreds of millions of daily active users and broad population reach, these short-video platforms have become major gateways for health information.^[12]^ Their features-rapid dissemination, wide coverage, engaging formats, strong interactivity, and low distribution costs-facilitate the communication of complex medical concepts in accessible ways, shorten the knowledge translation pathway, and enhance disease awareness and care-seeking intentions. Algorithmic delivery and social interaction can further amplify the visibility of high-quality content, promoting continuous iteration of science communication and audience segmentation.^[13]^ However, the rapid dissemination and wide reach of short-video platforms are also accompanied by potential risks, including uneven information quality, insufficient scientific rigor, and oversimplification of content. Previous studies on videos related to pancreatic cancer^[14]^ and hyperthyroidism^[15]^ have shown that, despite high engagement metrics, their overall quality and reliability are generally suboptimal, with frequent deficiencies in content completeness and evidence-based support. Such content can distort understanding, encourage inappropriate self-management or delay appropriate care, lengthen diagnostic and treatment pathways, increase redundant testing and ineffective interventions, and ultimately raise both individual economic burden and system-level healthcare costs.^[16]^ Given the high prevalence of hypothyroidism, the dissemination of inaccurate or insufficient health information may further exacerbate its burden. Therefore, it is essential to systematically evaluate the quality and reliability of hypothyroidism-related content on short-video platforms.

This study aims to evaluate the content, quality, and reliability of hypothyroidism-related videos on TikTok and Bilibili. By providing evidence-based and systematic analyses, the study seeks to promote improvements in health information quality, optimize platform governance and regulatory strategies, and ultimately enhance the effectiveness of health communication.

## 2. Materials and methods

### 2.1. Ethical considerations

This study did not involve clinical data, human specimens, or experimental animals. All study materials were derived from publicly accessible videos on TikTok and Bilibili, with strict compliance to privacy protection principles. The procedures for data collection and analysis fully adhered to the platforms’ terms and conditions. As there was no direct interaction with users, ethical approval and trial registration were not required.

### 2.2. Video collection method

**Figure 1.**
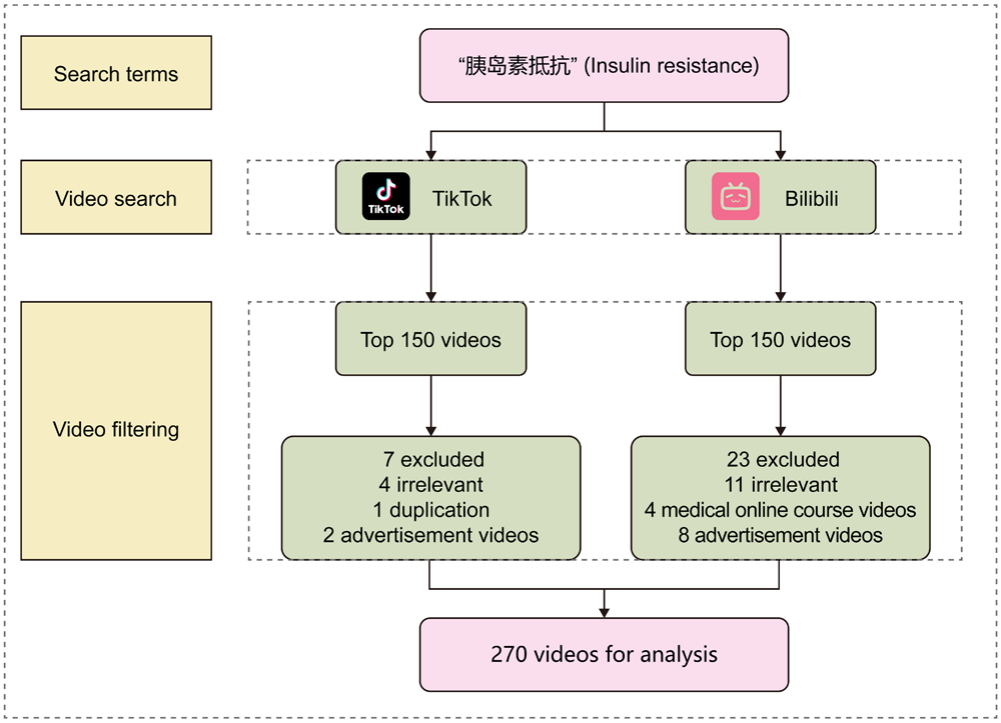
Flowchart of video selection and analysis process.

This study employed a cross-sectional design to systematically gather short-video data concerning “甲减” (a Chinese term for hypothyroidism, which is preferred by Chinese doctors and patients in their communication) on the platforms Bilibili and TikTok from September 13 to September 16, 2025. To mitigate the influence of personalized recommendation algorithms, searches were conducted while logged out. Each platform used the top 150 Chinese-language videos in the default comprehensive ranking as the initial sample for further screening, with the following exclusion criteria: videos not related to the topic; videos with duplicate content; videos for advertisement or commercial purposes; medical online course videos. A detailed collection method is depicted in Figure 1. For each included video, the following features were extracted: video length (seconds), number of likes, number of collections, number of comments, number of shares, uploader type, and content theme category (epidemiology, etiology, symptoms, diagnosis, treatment, prevention).

### 2.3. Uploader characteristics

The uploaders of the videos were categorized as: professionals and individual users. Professionals were further divided into: specialists, specifically endocrinologists and thyroid surgeons; and non-specialists, including practitioners of traditional Chinese medicine, nuclear medicine physicians, and other internal medicine or surgical specialists. Individual users consisted mainly of patients and health communicators.

### 2.4. Quality and reliability assessment

To evaluate the quality and reliability of short videos, this study employed 2 scoring tools: the Global Quality Score (GQS)^[17,18]^ and the modified DISCERN (mDISCERN)^[17,19]^ score. These tools are widely recognized for assessing medical and health information. The GQS is a widely used tool for assessing the quality of health information in videos, with response selection based on a 5-point scale from 1 (poor quality) to 5 (good quality). The mDISCERN score, initially designed for written health information, has been modified to assess video content reliability. It uses a 5-question format, with scores from 0 to 5, where higher scores indicate greater reliability. Detailed scoring criteria are provided in Tables 1 and 2. To ensure methodological rigor, a double-blind evaluation procedure was employed, whereby each rater conducted assessments independently without access to the other’s scores during the initial evaluation. In instances of disagreement, a 3rd reviewer was consulted to adjudicate and reach consensus. Prior to scoring, all assessors received uniform training to ensure consistency of scoring and to minimize bias.

**Table 1:** The Global Quality Score (GQS) quality criteria.

Item features	Points
Poor quality; poor flow of the videos; most information missing; not at all useful for patients	1
Generally poor quality; some information listed, but many important topics missing; of very limited use to patients	2
Moderate quality; suboptimal flow; some important adequately discussed, but other information poorly discussed; somewhat useful for patients	3
Good quality and generally good flow; most of the relevant information listed, but some topics not covered; useful for patients	4
Excellent quality and flow; very useful for patients	5

**Table 2:** The modified DISCERN (mDISCERN) quality criteria.

Reliability score
1. Is the video clear, concise, and understandable?
2. Are valid sources cited?
3. Is the content presented balanced and unbiased?
4. Are additional sources of content listed for patient reference?
5. Are areas of uncertainty mentioned?

### 2.5. Statistical analysis

Descriptive statistics were used to summarize video characteristics, with continuous variables expressed as the median and interquartile range (IQR) and categorical variables expressed as frequency and percentage. Comparisons between the 2 platforms were performed using the Mann–Whitney *U* test, and comparisons among different uploader groups were conducted using the Kruskal–Wallis test. Inter-rater reliability was assessed using Cohen kappa statistic. A Cohen kappa value of ≥0.75 was considered to indicate good agreement between raters.

The correlation between video engagement metrics and quality/reliability scores was analyzed using Spearman rank correlation coefficient. Statistical analyses were performed in R (version 4.3.2; R Foundation for Statistical Computing, Vienna, Austria), and a 2-sided *P* value < .05 was considered statistically significant.

## 3. Results

### 3.1. Video characteristics by platform

**Figure 2.**
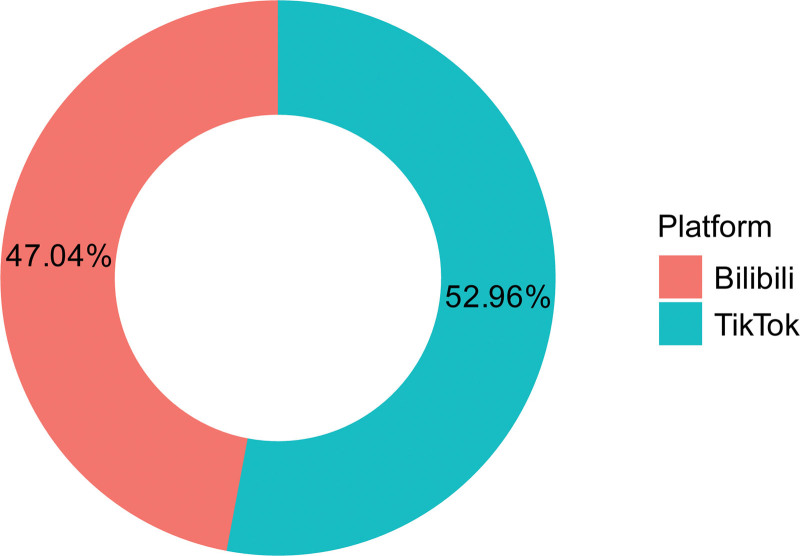
Distribution of video platforms.

Based on the predefined inclusion and exclusion criteria, we analyzed 270 videos from Bilibili and TikTok, of which 127 (47.04%) were from Bilibili and 143 (52.96%) were from TikTok (Fig. 2). As shown in Table 3, among 270 videos, the median length was 80.50 seconds (IQR: 49.50–160.50 seconds). The median numbers of likes, collections, comments, and shares were 174.50 (IQR: 35.25–1152.50), 91.50 (IQR: 19.25–587.75), 15.00 (IQR: 3.00–125.00), and 61.00 (IQR: 11.00–450.00), respectively. Content primarily addressed treatment 168 (62.2%) and symptoms 162 (60.0%), with less emphasis on etiology 92 (34.1%) and diagnosis 73 (27.0%), while prevention 19 (7.0%) and epidemiology 11 (4.1%) were least represented. Median quality and reliability were modest, with GQS 2.00 (IQR: 1.00–3.00) and mDISCERN 2.00 (IQR: 1.00–3.00). Compared with TikTok, videos on Bilibili were significantly longer in duration but received fewer likes, collections, comments, and shares (all *P* < .05). The TikTok group had higher mDISCERN scores than the Bilibili group, with a statistically significant difference, while no significant difference in GQS scores was observed between the 2 groups (Table 4).

**Table 3:** General characteristics, quality, and reliability of the videos.

Variables	Total (n = 270)
General information	
Video length(s), median (IQR)	80.50 (49.50–160.50)
Likes, median (IQR)	174.50 (35.25–1152.50)
Collections, median (IQR)	91.50 (19.25–587.75)
Comments, median (IQR)	15.00 (3.00–125.00)
Shares, median (IQR)	61.00 (11.00–450.00)
Video content	
Epidemiology, n (%)	11 (4.1%)
Etiology, n (%)	92 (34.1%)
Symptoms, n (%)	162 (60.0%)
Diagnosis, n (%)	73 (27.0%)
Treatment, n (%)	168 (62.2%)
Prevention, n (%)	19 (7.0%)
Video quality	
GQS score, median (IQR)	2.00 (1.00–3.00)
mDISCERN score, median (IQR)	2.00 (1.00–3.00)

GQS = Global Quality Score, IQR = interquartile range, mDISCERN = modified DISCERN.

**Table 4:** General characteristics, quality, and reliability by platform.

Variables	Bilibili (n = 127)	TikTok (n = 143)	*P* value
Video length (s), median (IQR)	105.00 (54.00–269.00)	67.00 (48.00–105.50)	<.001
Likes, median (IQR)	33.00 (5.00–125.00)	816.00 (190.00–1980.50)	<.001
Collections, median (IQR)	25.00 (2.00–109.50)	372.00 (73.00–1352.50)	<.001
Comments, median (IQR)	4.00 (0.00–17.50)	60.00 (10.00–286.50)	<.001
Shares, median (IQR)	11.00 (1.00–47.00)	355.00 (60.00–1093.00)	<.001
GQS, median (IQR)	2.00 (1.00–3.00)	2.00 (1.00–3.00)	.057
mDISCERN, median (IQR)	2.00 (0.00–2.00)	2.00 (2.00–3.00)	<.001

GQS = Global Quality Score, IQR = interquartile range, mDISCERN = modified DISCERN.

### 3.2. Video characteristics by uploader type

**Figure 3.**
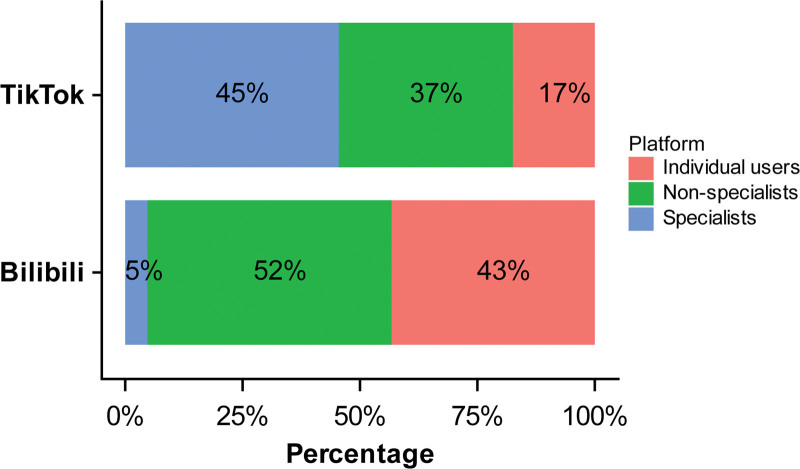
Distribution of video uploaders on Bilibili and TikTok.

Among the 270 videos, 119 (44.1%) were uploaded by non-specialists, 80 (29.6%) by individual users, and 71 (26.3%) by specialists. Across platforms, on Bilibili, 52.0% of videos were uploaded by non-specialists, 43.3% by individual users, and 4.7% by specialists; on TikTok, 45.5% were uploaded by specialists, 37.1% by non-specialists, and 17.5% by individual users (Fig. 3). Median video length varied by uploader type: individual users, 185.00 seconds (IQR: 97.50–324.25 seconds); non-specialists, 63.00 seconds (IQR: 45.50–104.00 seconds); and specialists, 60.00 seconds (IQR: 45.00–103.50 seconds). Although videos uploaded by individual users had the longest median length, those from specialists achieved the highest engagement, with median values of 436 likes (IQR: 123.00–1487.50), 221 collections (IQR: 35.50–1010.00), 31 comments (IQR: 9.00–209.00), and 255 shares (IQR: 30.00–772.50). All between-group differences in video characteristics were statistically significant (*P* < .05) (Table 5).

**Table 5:** Comparison of characteristics, quality, and reliability across different uploader types.

Variables	Individual users (n = 80)	Non-specialists (n = 119)	Specialists (n = 71)	*P* value
Video length (s), median (IQR)	185.00 (97.50–324.25)	63.00 (45.50–104.00)	60.00 (45.00–103.50)	<.001
Likes, median (IQR)	158.50 (55.00–833.50)	87.00 (10.00–851.00)	436.00 (123.00–1487.50)	<.001
Collections, median (IQR)	124.50 (33.00–568.00)	51.00 (4.50–404.00)	221.00 (35.50–1010.00)	.011
Comments, median (IQR)	19.00 (4.00–271.00)	6.00 (0.00–45.50)	31.00 (9.00–209.00)	<.001
Shares, median (IQR)	47.50 (12.75–352.00)	39.00 (5.00–288.00)	255.00 (30.00–772.50)	.002
GQS, median (IQR)	2.00 (1.00–3.00)	2.00 (1.00–2.00)	3.00 (2.00–3.00)	<.001
mDISCERN, median (IQR)	1.00 (1.00–2.00)	2.00 (1.00–2.00)	2.00 (2.00–3.00)	<.001

GQS = Global Quality Score, IQR = interquartile range, mDISCERN = modified DISCERN.

### 3.3. Video content analysis

**Figure 4.**
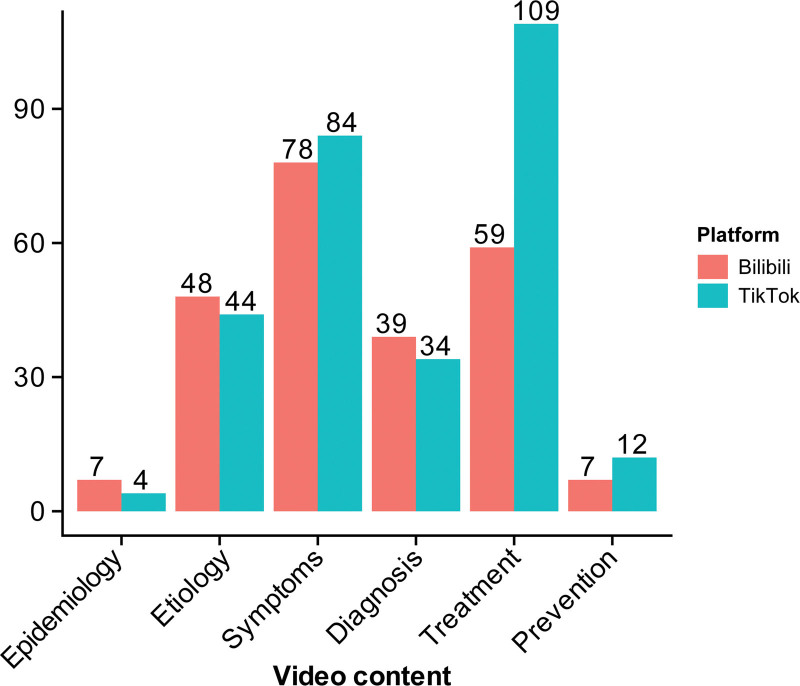
Distribution of video content on Bilibili and TikTok.

Of the 270 videos, those mentioning treatment and symptoms accounted for the largest proportions, with counts of 168 (62.2%) and 162 (60.0%), respectively. The number of videos mentioning treatment was markedly higher on TikTok than on Bilibili. Videos mentioning causes and diagnosis numbered 92 (34.1%) and 73 (27.0%), respectively. Content related to prevention and epidemiology was scarce, accounting for only 19 (7.0%) and 11 (4.1%) videos, respectively. Notably, only 4 of the 143 TikTok videos mentioned epidemiology (Fig. 4).

### 3.4. Video quality

**Figure 5.**
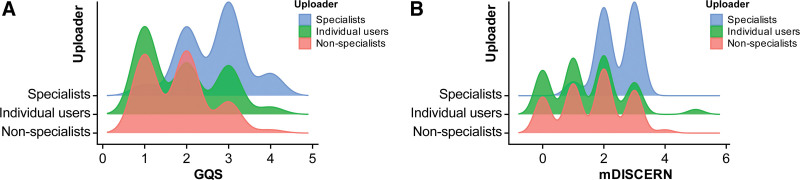
Distribution of video quality scores across different uploaders. (A) Distribution of GQS across different uploaders. (B) Distribution of mDISCERN across different uploaders. GQS = Global Quality Score, mDISCERN = modified DISCERN.

**Figure 6.**
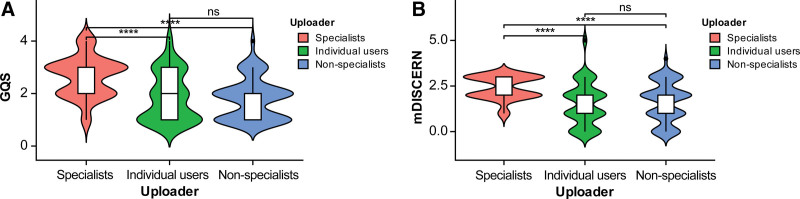
Comparison of video quality scores across different uploaders. (A) Comparison of GQS across different uploaders. (B) Comparison of mDISCERN across different uploaders. ****P < .0001; ns = non-significant. GQS = Global Quality Score, mDISCERN = modified DISCERN.

The inter-rater agreement for GQS and mDISCERN scores was excellent, with Cohen kappa values of 0.804 and 0.782, respectively. As shown in Table 3, the median GQS score was 2.00 for both Bilibili and TikTok (IQR: 1.00–3.00 for each), with no statistically significant difference (*P *> .05). For mDISCERN, the median score was 2.00 (IQR: 0.00–2.00) for Bilibili and 2.00 (IQR: 2.00–3.00) for TikTok, indicating a statistically significant difference (*P* < .05). Figure 5A and B shows the distributions of GQS and mDISCERN scores among different uploaders, respectively. Furthermore, as illustrated in Figure 6A and B, violin plots revealed that specialists outperformed other groups in both GQS and mDISCERN with statistically significant differences, whereas no significant differences were observed between non-specialists and individual users in either metric.

### 3.5. Correlation analysis

**Figure 7.**
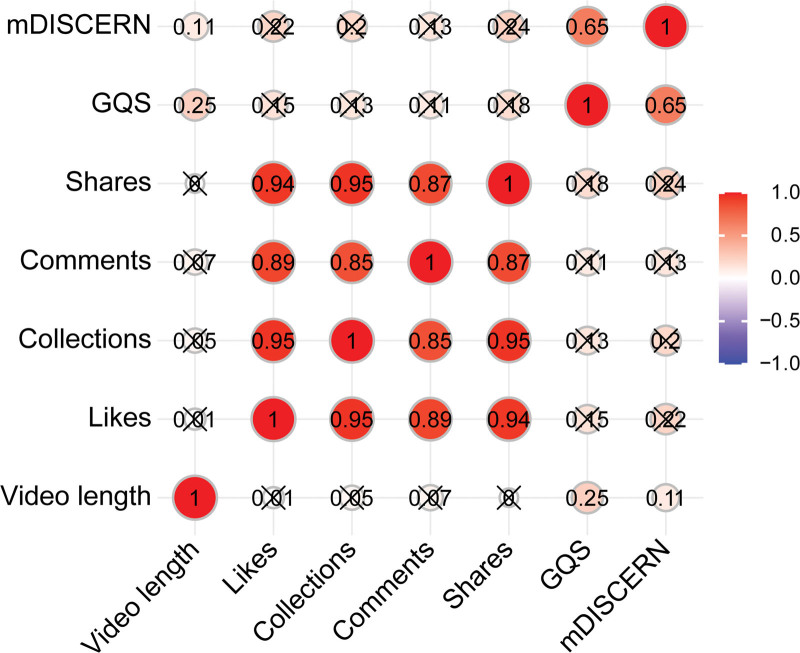
Spearman correlation analysis among different video variables, GQS, and mDISCERN score. GQS = Global Quality Score, mDISCERN = modified DISCERN.

Spearman correlation analysis was conducted to examine associations between engagement metrics (likes, comments, collections, and shares) and GQS and mDISCERN scores. As shown in Figure 7, interactive metrics were strongly and positively correlated with each other; for example, the correlation coefficients for likes with collections, comments, and shares were 0.95, 0.89, and 0.94, respectively. In contrast, the engagement metrics did not show a statistically significant correlation with GQS and mDISCERN scores. Video length showed a weak correlation with GQS and mDISCERN scores, with correlation coefficients of 0.25 and 0.11, respectively.

## 4. Discussion

This study evaluated 270 short videos on hypothyroidism from Bilibili and TikTok. Videos on Bilibili were longer in duration but exhibited lower engagement. TikTok videos, however, showed higher mDISCERN scores. The most common topics in the videos were treatment and symptoms, while prevention and epidemiology were underrepresented. Videos uploaded by specialists received higher GQS and mDISCERN scores. Additionally, engagement metrics were highly intercorrelated but showed no association with quality scores. Video length exhibited only a weak correlation with quality. This study revealed significant differences in the quality and reliability of hypothyroidism-related videos, particularly the neglect of prevention and epidemiology content. This underscores the need for enhanced supervision and improvement of health information quality on short-video platforms to ensure that the public receives more scientifically accurate and reliable health information.

Content analysis showed that treatment and symptom topics were most common, while etiology and diagnosis appeared less often. More importantly, prevention and epidemiology were very scarce (on TikTok, epidemiology was only mentioned in a few cases). This may be because epidemiology and prevention are technical and less engaging, so they struggle to gain attention on short-video platforms. Large-scale epidemiological surveys indicate a very high underdiagnosis rate of hypothyroidism, with rates exceeding 90% in certain populations.^[20]^ While focusing solely on treatment and clinical presentation, neglecting epidemiological data and preventive measures, would lead to substantial underdiagnosis, delayed treatment, increased risk of complications, and a heightened public health burden.^[21]^ Conversely, emphasizing prevention can markedly reduce both incidence and disease burden. Epidemiological factors such as iodine nutrition status, environmental triggers, and genetic susceptibility are critical to the prevention of hypothyroidism. For example, implementing iodized salt programs in iodine-deficient regions can effectively reduce the incidence of hypothyroidism^[21]^; similarly, primary prevention strategies such as newborn screening can prevent irreversible neurological damage caused by congenital hypothyroidism.^[22]^ Other studies report similar patterns: in a study of *Helicobacter pylori* videos, household infection control and management accounted for only 5.8%, with even less on prevention^[23]^; in fire and burn prevention videos, coverage of the 15 World Health Organization-recommended measures ranged from 0% to 77.86%,^[24]^ and some key measures were never mentioned; health videos on YouTube also show insufficient coverage of preventive behaviors.^[25,26]^ This imbalance entails 3 potential risks. First, an excessive focus on treatment may diminish attention to primary and secondary prevention, long-term follow-up, and lifestyle management.^[23]^ Second, the absence of an epidemiological context can weaken population-level risk awareness and obscure care pathways, thereby hindering early screening and diagnosis. Finally, symptom-and therapy-related information often appears fragmented, lacking incidence data, risk factor analysis, and evidence grading, which may contribute to nonspecific anxiety or overtreatment.^[27]^ We recommend “breaking it into small pieces”: use micro-series and structured info cards to present prevention and epidemiology in short, digestible units, include clear calls to action, and link to authoritative sources to reduce the risk of misinformation.

Platform comparisons indicate that Bilibili hosts longer videos but achieves lower engagement, whereas TikTok, despite its shorter video durations, exhibits markedly higher levels of engagement, a pattern consistent with findings from other studies.^[28]^ This may be because shorter videos tend to be more addictive and spread more rapidly.^[29]^ In terms of reliability and overall quality, there was no significant difference in GQS between platforms, whereas TikTok showed higher mDISCERN scores. This suggests a divergence between performance on information completeness/source disclosure (mDISCERN) and perceived overall quality (GQS). A plausible mechanism is that TikTok favors concise, bullet-point delivery that facilitates certain mDISCERN criteria (e.g. clarity and provision of additional sources) without necessarily improving overall perceived quality^[28]^; by contrast, Bilibili longer format supports comprehensive explanations but suffers from low completion rates and slower diffusion, which depress engagement.^[30–32]^ Consequently, e-health strategies should be platform-specific: on TikTok, use high-density content with a clear evidence chain to strengthen credibility, whereas on Bilibili, implement chapters, progress markers, and navigation to structure long videos for skimming and retrieval, mitigating the risks of “long but shallow” or “too long to finish.”

Analysis by uploader type indicates that although specialists uploaded the shortest videos, they outperformed non-specialists and individual users on both engagement metrics and GQS and mDISCERN scores. This finding aligns with conclusions drawn by similar studies.^[23,33,34]^ In contrast, GQS and mDISCERN scores did not show significant differences between non-specialists and individual users. These patterns may be explained by 2 factors: specialist credentials serve as a “trust pass,” making audiences more receptive and more likely to engage; and specialists, owing to their comprehensive professional knowledge, tend to achieve higher information delivery efficiency, conveying key information clearly within shorter time frames. Regrettably, our results show that specialists account for 45.5% of health-related videos on TikTok but only 4.7% on Bilibili. Therefore, we recommend that platforms encourage greater participation of specialists in health education content creation and increase the provision of standardized content aligned with clinical guidelines to enhance the scientific quality and impact of health information dissemination. Platforms could enhance the visibility of specialist videos through algorithmic prioritization and homepage featuring, thereby incentivizing physicians to engage in sustained content creation.

Furthermore, Spearman correlation analysis showed no association between engagement metrics and either GQS or mDISCERN. These results are consistent with findings reported in prior studies. A study that analyzed 164 knee osteoarthritis-related videos on TikTok and Bilibili found that although videos produced by professional institutions achieved the highest quality and reliability scores, engagement metrics were not correlated with video quality.^[35]^ Another study on gallstone-related videos on TikTok similarly revealed that while most videos were uploaded by doctors and primarily focused on disease knowledge, the overall quality remained low, with high-quality videos receiving limited attention and popular videos generally lacking reliability.^[36]^ This indicates that the professionalism and scientific rigor of videos do not directly determine their popularity. Previous studies have found that short video platform users tend to prefer content that is entertaining and attention-grabbing. High-credibility professional videos may appear dull to audiences due to their lack of entertainment value, making it harder for them to gain traction.^[37]^ Videos with misleading or incomplete content sometimes gain more popularity instead.^[38]^ Therefore, it is recommended that platforms and content creators enhance narrative expressions to increase the fun and appeal of the content, thereby achieving more effective health communication. Simultaneously, embedding authoritative links and recommending professional websites within entertaining content can help users access more comprehensive and reliable health knowledge.^[26,39]^ Spearman correlation analysis revealed very high correlations among engagement metrics such as likes, shares, and collections, indicating that users’ multidimensional recognition behaviors exhibit consistency. This high correlation implies that a single engagement metric (e.g., likes) can to some extent represent overall engagement intensity, enabling platforms to optimize content recommendations and popularity ranking algorithms accordingly.^[40,41]^

This study has several limitations that should be taken into account when interpreting the findings.

First, this research employed a cross-sectional design, which can only reflect the quality of videos at a specific time point and cannot capture temporal variations in content. Future longitudinal or periodic follow-up studies are needed to explore the dynamic evolution of health information quality. Second, the dynamic nature of platform algorithms may affect the reproducibility of results. As TikTok and Bilibili continuously update their recommendation mechanisms and video rankings, search results may vary over time or by user characteristics, introducing potential retrieval bias. Third, although this study analyzed 270 videos from 2 major Chinese platforms, the sample size remains limited relative to the vast amount of online content, and the narrow platform scope may restrict the generalizability and external validity of the findings. Fourth, the assessment of video quality and reliability relied mainly on manual evaluation. Although multiple validated tools were used and double-blind rating with 3rd-party adjudication was implemented to reduce bias, a certain degree of subjectivity could not be completely avoided. Finally, this study included only Chinese-language videos; therefore, the findings may not be fully generalizable to other linguistic or cultural contexts. Future research should incorporate multilingual and cross-cultural platforms to enhance international applicability and external validity.

## 5. Conclusion

This study evaluated the content, quality, and reliability of hypothyroidism-related videos on TikTok and Bilibili. The content structure of these videos was incomplete, with insufficient emphasis on prevention and epidemiology. The quality and reliability of the videos were suboptimal. Videos uploaded by specialists had the highest quality and reliability. Future efforts should encourage greater involvement of specialists in hypothyroidism-related health education, strengthen platform monitoring of content and quality, and optimize video recommendation algorithms to ensure that viewers receive more comprehensive and reliable information.

## Acknowledgments

We acknowledge TikTok and Bilibili as sources of publicly accessible data. Any opinions expressed are solely those of the authors.

## Author contributions

**Conceptualization:** Zongyou Cheng.

**Formal analysis:** Yaqin Hu.

**Investigation:** Zongyou Cheng.

**Methodology:** Yaqin Hu.

**Project administration:** Hongmin Zhu.

**Supervision:** Hongmin Zhu.

**Writing – original draft:** Yaqin Hu.

**Writing – review & editing:** Zongyou Cheng, Hongmin Zhu.
